# Quantitative Detection of Cracks in Steel Using Eddy Current Pulsed Thermography

**DOI:** 10.3390/s18041070

**Published:** 2018-04-02

**Authors:** Zhanqun Shi, Xiaoyu Xu, Jiaojiao Ma, Dong Zhen, Hao Zhang

**Affiliations:** School of Mechanical Engineering, Hebei University of Technology, Tianjin 300130, China; z_shi@hebut.edu.cn (Z.S.); XiaoyuXu0701@163.com (X.X.); majiaojiaos@163.com (J.M.); zhanghao@hebut.edu.cn (H.Z.)

**Keywords:** ECPT, quantitative detection, cracks, temperature variation

## Abstract

Small cracks are common defects in steel and often lead to catastrophic accidents in industrial applications. Various nondestructive testing methods have been investigated for crack detection; however, most current methods focus on qualitative crack identification and image processing. In this study, eddy current pulsed thermography (ECPT) was applied for quantitative crack detection based on derivative analysis of temperature variation. The effects of the incentive parameters on the temperature variation were analyzed in the simulation study. The crack profile and position are identified in the thermal image based on the Canny edge detection algorithm. Then, one or more trajectories are determined through the crack profile in order to determine the crack boundary through its temperature distribution. The slope curve along the trajectory is obtained. Finally, quantitative analysis of the crack sizes was performed by analyzing the features of the slope curves. The experimental verification showed that the crack sizes could be quantitatively detected with errors of less than 1%. Therefore, the proposed ECPT method was demonstrated to be a feasible and effective nondestructive approach for quantitative crack detection.

## 1. Introduction

Steel is a widely used metal in industry and plays a vital role in the aerospace, transportation, weapon, and energy fields [1]. Cracks are some of the most serious defects in steel structures, because they tend to grow under stress [2]. It is common for cracks to originate at the surface during production and use, which will directly affect the use and service performance of the entire mechanical system. Small cracks often cause large accidents in engineering, resulting in significant economic losses and casualties. Therefore, the development of crack detection methods holds great significance for improving the reliability of equipment and preventing the occurrence of catastrophic accidents.

As one of the basic technologies for crack detection and identification, nondestructive testing has been playing an increasingly important role in ensuring the quality of products and engineering structures. These approaches avoid destruction of the internal structure of the objects and instead rely on physics-based methods to determine the surface and internal properties and to verify whether internal discontinuities (defects) are present. These techniques can be used to judge whether the object is qualified and evaluate its applicability [3,4,5,6,7]. Nondestructive testing systems were mainly established in the middle of the 20th century and are represented by five general testing technologies: radiographic testing, ultrasonic testing, magnetic testing, penetration testing, and eddy current testing [8]. Currently, conventional nondestructive testing techniques used for crack detection on metal surfaces include magnetic testing, penetration testing, and eddy current testing. Each of these testing technologies has unique advantages, as well as certain limitations [9]. Magnetic testing is only suitable for crack detection in ferromagnetic materials. Penetration testing can be used to detect cracks in metallic and non-metallic materials but is not suitable for the detection of small cracks. Eddy current testing can be used to determine whether a defect is present but cannot provide a rating on the shape or size of the defect. Therefore, there is a great practical need to develop a method that allows quantitative determination of the size of small cracks in metals.

Eddy current pulsed thermography (ECPT) [10,11,12] is a recently developed nondestructive testing method that combines eddy current testing and infrared thermography. The quality, internal state, structure, and defects of the examined objects are determined based on the temperature field distribution. From the light and dark areas in the thermal image, the location of defects can be determined [13]. Compared with conventional nondestructive testing methods, this approach offers many advantages as it is a non-contact measurement technique with high sensitivity, fast response, and a large detection area that provides intuition and high precision [14]. Therefore, ECPT has been studied and rapidly developed in various countries and has been used to evaluate metals and composites [15].

Using simulations and experiments, Oswald and Tian analyzed the surface defects of metallic materials using ECPT to investigate the effect of the eddy penetration depth on the temperature [16]. Weekes investigated the smallest flaws detectable by ECPT in alloys such as steel and nickel [17]. Matthias et al. studied the relationship between the temperature and the depth and width of cracks under ANSYS simulation conditions and discussed the feasibility of quantitative detection [18]. Cheng et al. built an ECPT detection system that could detect layered defects with depths of less than 1 mm [19]. Yang, Tian, and Ilham studied the problems associated with detecting cracks at metal edges [20]. Ilham and Tian considered the capabilities of ECPT for obtaining quantitative information about cracks with an angle on the surface [21]. He and Tian applied ECPT to common metal cracks and analyzed the relationship between the crack depth and temperature change [22,23].

Most research on flaw detection technology based on ECPT has involved qualitative analysis of cracks and processing of infrared thermal images, and the approaches could only be used to identify and locate cracks. However, quantitative analysis of the crack size has not yet been investigated in depth. Therefore, the main target of this study was to develop a quantitative evaluation method for crack size detection based on ECPT technology.

The remainder of this paper is organized as follows. Section 2 discusses the theoretical basis of the ECPT detection technology and the process of quantitative analysis methods. Section 3 introduces the simulation and analysis of the cracked model. The best parameters were determined, and the effects of the cracks on the eddy current field and temperature field distribution were explored. The simulation verified the quantitative evaluation method. The experimental study is covered, and a discussion of the results is presented in Section 4; the quantitative crack evaluation method is also presented in this section. Finally, the conclusions and future work are discussed in Section 5.

## 2. Detection Principle and Method

**Figure 1:**
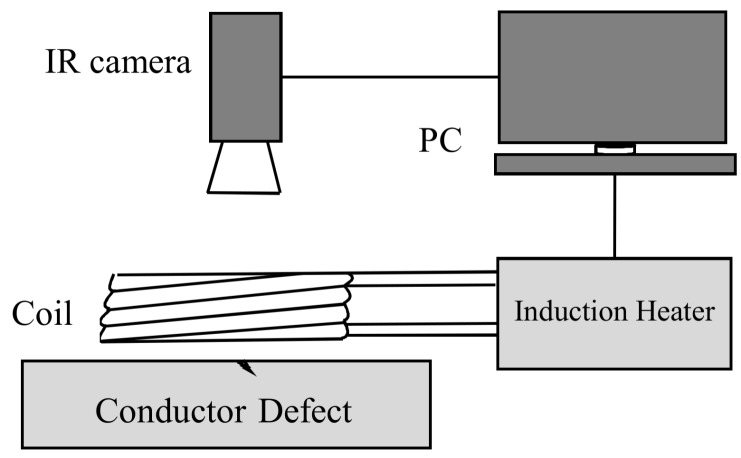
Schematic diagram of ECPT.

The configuration of the ECPT system [24] is shown in Figure 1.

The working principle of the system is that the alternating magnetic field is generated by the high-frequency current in the coil, which produces an induced current in the sample. The eddy current is forced to bypass the crack when the sample is flawed, leading to an increase or decrease of the eddy current density. Based on Joule’s law, the distribution of the heat produced in the conductor will be uneven; thus, the heat generated at the surface of the conductor is captured by an infrared camera with the data stored on a PC.

### 2.1. Eddy Current Field

According to the law of electromagnetic induction, there is an alternating magnetic field near a wire with alternating current, and an eddy current will be produced in a conductor placed in the magnetic field. According to Maxwell’s equations [25],

(1) $$\{\begin{array}{c} \nabla \times \bar{H}=\bar{J}+\frac{\partial \bar{D}}{\partial t}=\bar{J_{s}}+\bar{J_{e}}+\frac{\partial \bar{D}}{\partial t},\\ \nabla \times \bar{E}=-\frac{\partial \bar{B}}{\partial t} \end{array}$$

where H is the magnetic field intensity, D is the electric potential shift vector, E is the electric field intensity, B is the magnetic flux density, J is the total current density, $J_{s}$ is the external current density, and $J_{e}$ is the induced current density.

Based on the partial differential equation and vector operation, the following control equation for the eddy current field is obtained:

(2) $$\nabla \times \left(\frac{1}{\mu }\nabla \times \bar{A}\right)+j\omega \sigma \bar{A}-\omega^{2}\varepsilon \bar{A}=\bar{J_{s}},$$

in which μ is the magnetic permeability (H/m), $\bar{A}$ is the vector magnetic potential, σ is the electrical conductivity (S/m), and ε is the dielectric constant.

The conductivity and permeability of the conductor will change when cracks are present in the conductors, such that the eddy current distribution in the conductor will also change.

### 2.2. Temperature Field

As a heat source, Joule heat produced by an eddy current can heat materials; therefore, the temperature distribution is determined by the eddy current distribution and heat transfer in conductors. The penetration depth of the eddy current is called the skin depth δ:

(3) $$\delta =\frac{1}{\sqrt{\pi \mu \sigma f}},$$

in which f is the current frequency.

Coupling the eddy current field and temperature field using Joule’s law, the heating power produced by the eddy current in the conductor can be described by Q:

(4) $$Q=\frac{1}{\sigma }{\left|J_{e}\right|}^{2}=\frac{1}{\sigma }{\left|\sigma E\right|}^{2}.$$

Joule heat spreads in the solder ball inside following the propagation law

(5) $$\rho C_{P}\frac{\partial T}{\partial t}-\nabla \cdot k\nabla T=Q.$$

### 2.3. Detection Method

**Figure 2:**
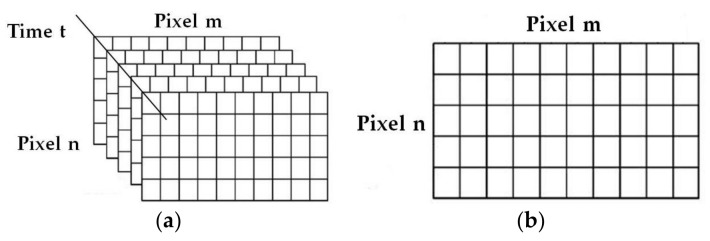
The data format diagram for ECPT: (**a**) sequence of infrared thermal image and (**b**) thermal image sequence at a certain time.

The raw data recorded by the thermal imager is a three-dimensional array when using ECPT to detect surface cracks of steel. As shown in Figure 2a, m and n are the number of pixels of the infrared camera, and t is the number of recorded times. The measured value of each time constitutes an m × n two-dimensional matrix, shown in Figure 2b, which is the thermal image of ECPT detection.

Canny edge detection algorithm [26] is a multi-level edge detection algorithm developed by John Canny in 1986. Canny transforms the edge detection problem into the maximum value of the detection unit function. The first-order differential of the Gaussian function is used in Canny edge detection method and has a good balance between noise suppression and edge detection. The two convolution operators of Canny algorithm are:

(6) $$S_{x}=\left(\begin{array}{cc} -1 & 1\\ -1 & 1 \end{array}\right),\;S_{y}=\left(\begin{array}{cc} 1 & 1\\ -1 & -1 \end{array}\right)$$

The first-order partial matrix in the *x*-axis P and *y*-axis directions Q is:

(7) P(i,j)=(f(i+1,j−1)+f(i+1,j)+f(i+1,j+1)−f(i+1,j)]/2

(8) Q(i,j)=(f(i,j)−f(i+1,j)+f(i,j+1)−f(i+1,j+1)]/2

The gradient magnitude is:

(9) $$M\left(i,j\right)=\sqrt{P{\left(i,j\right)}^{2}+Q{\left(i,j\right)}^{2}}$$

The gradient direction angle that represents the intensity of the edge data is:

(10) θ(i,j)=arctan[Q(i,j)/P[i,j])

The two-dimensional array of thermal images is processed by the Canny algorithm, and the crack boundary can be observed. According to the external shape of the crack edge, not only the basic shape of the crack can be determined, but also the position of the crack in the two-dimensional array can be roughly determined. In order to further analyze the size of the crack, one or more trajectories (straight line or curve) that are selected based on the shape of the crack through the crack area can be determined in the two-dimensional array.

The temperature distribution along the trajectory is analyzed and its slope curve is plotted according to Equation (11).

(11) $$k\left(i,j\right)=\frac{T_{i}-T_{j}}{i-j},$$

in which k is the slope between the pixels i to j, $T_{i}$ is the temperature at pixel i, and $T_{j}$ is the temperature at pixel j.

When the excitation signal and material properties are the same, the slope of the curve is only affected by the defect geometry. Therefore, the curve slope can be used for quantitative evaluation of cracks based on the accumulation of considerable experimental data.

## 3. Simulation Model and Method Verification

Numerical simulation is an effective tool to study the multi-physics of ECPT of conductive samples [27]. COMSOL Multiphysics is a 3D simulation platform used for building numerical models and analyzing induction heating and heat transfer processes. The simulation model consists of three parts with a four-turn coil, test piece, and shield. Through simulation of the defective sample, the effect of each parameter can be determined. The change law of the crack boundary is determined by analyzing the crack profile and the temperature of the trajectory, which provided a theoretical basis for the quantitative crack detection analysis in the experiment.

### 3.1. Simulation Modeling of Cracks

**Figure 3:**
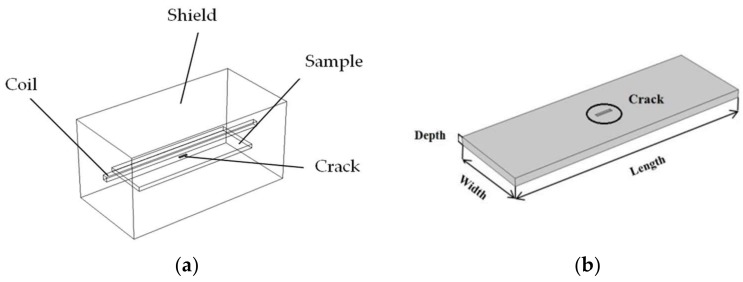
Simulation model for ECPT: (**a**) 3D model and (**b**) steel model.

The simulation model was built using the Induction Heating module of the COMSOL platform. The study concentrated on rectangular cracks on the surface of steel samples. As shown in Figure 3a, the four-turn straight coil was a copper inductor with a diameter of 6 mm and a length of 200 mm, which was activated by a high-frequency impulse signal. The sample size was 150 mm × 50 mm × 5 mm. A rectangular crack with a size of 10 mm × 2 mm × 0.6 mm was located on the surface of the sample. The shield size was 200 mm × 150 mm × 100 mm. The material properties used in the simulation are listed in Table 1.

### 3.2. Effect of Incentive Parameters

**Figure 4:**
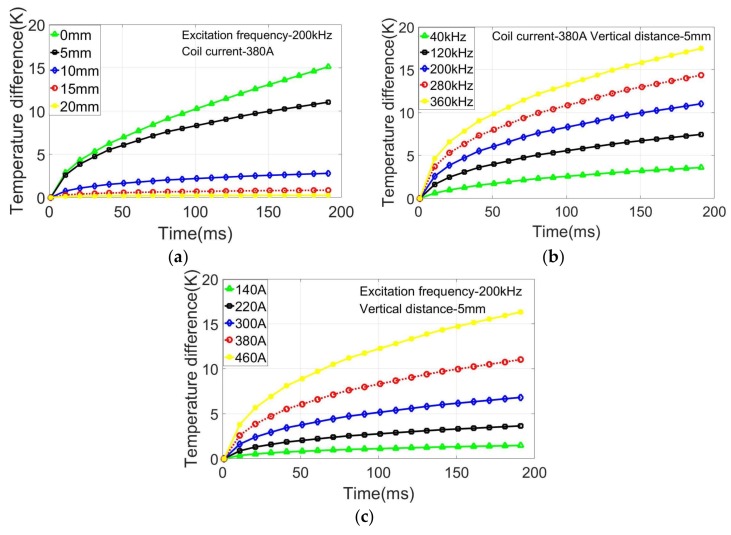
Temperature variation as a function of (**a**) vertical distance, (**b**) excitation frequency, and (**c**) coil current.

To optimize the incentive parameters for accurate detection results in the experimental studies, the effects of the coil current, excitation frequency, heating time, and vertical distance between the coil and sample on the temperature variation were investigated in the simulation studies. According to the limitations of the experimental equipment and the experimental results compared with the previous experiments, the basic incentive parameters of the model were as follows: the vertical distance was 5 mm, the coil current was 380 A, and the excitation frequency was 200 kHz. The simulation models were established using different vertical distances, coil currents, and excitation frequencies with the other parameters fixed. The effects were verified by analyzing the temperature difference between two points in the defect and non-defect areas. The temperature variations at different times and under different conditions are shown in Figure 4.

The temperature variation as a function of vertical distance is shown in Figure 4a; the maximum distance of the experimental device was 20 mm, and the other distances were obtained by reducing this distance to 15, 10, 5, and 0 mm. The temperature difference was the largest for a distance of 0 mm and second-largest for a distance of 5 mm. However, the distance in the experiment cannot be 0 mm because of the isolation of the coil. The temperature difference was close to 0 K when the distance was greater than 20 mm. These results indicate that the temperature difference gradually increased with decreasing distance, but that beyond a critical value of the vertical distance, there was no effect on the temperature variation. Reducing the vertical distance between the coil and sample can also increase the heating efficiency. However, due to the proximity effect, the current distribution will be more concentrated when the distance is small, resulting in a smaller heating range. In the experiment, it is often necessary to select an appropriate vertical distance according to the crack condition so as to obtain a large heating range, as well as to ensure the heating effect.

The temperature variations as a function of the coil current and excitation frequency are shown in Figure 4b,c, respectively. The highest excitation frequency and coil current were 360 kHz and 460 A, respectively. The temperature difference greatly increased with increasing coil current and excitation frequency with regularity. The results show that the heating effect is affected by the current and frequency. The excitation parameters that are selected should be controlled within a certain range, due to the limitation of the excitation parameters for the induction heating device. As shown in Figure 4, the heating time also affected the heating effect. The temperature difference gradually increased with increasing heating time and rapidly increased at the beginning of the heating.

It can be concluded that reducing the vertical distance between the coil and sample surface and increasing the coil current and excitation frequency can improve the heating efficiency of the coil. The influencing law of the parameters was used in the experiment based on the actual conditions, and the appropriate parameters were selected to ensure a better heating effect.

### 3.3. Simulation Analysis and Method Verification of Cracks

**Figure 5:**
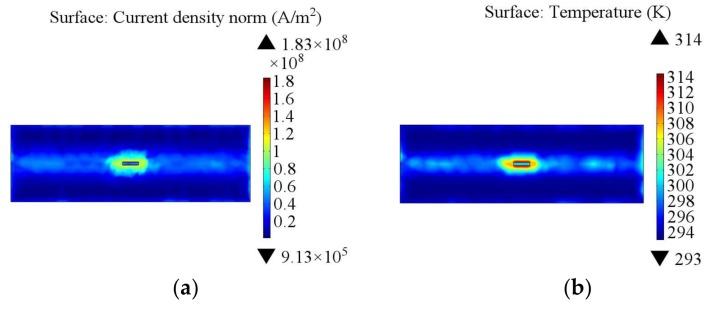
Simulation images of surface crack: (**a**) surface current density (A/m^2^) and (**b**) surface temperature (K).

The appropriate parameters were selected based on the incentive parameters analysis when the simulation model was established. The vertical distance between the coil and steel sample was set to 5 mm with a coil current of 380 A and an excitation frequency of 200 kHz. Under the same conditions, the effect of the cracks on the distribution of the eddy current and temperature after a short period of 200 ms for the induction heating are shown in Figure 5. The coupling effect was analyzed and discussed in combination with the induced eddy current and heat distribution for the crack evaluation.

As shown in Figure 5a, a number of eddy currents converged in the crack boundaries, resulting in a larger eddy current density in the boundaries. In addition, a higher temperature was produced in the crack boundaries, as shown in Figure 5b. The temperature distribution was consistent with the current density distribution, with positions with large current density corresponding to high temperature. These phenomena indicate that the crack caused variation of the eddy current distribution in the sample, leading to a buildup of eddy currents and temperature near the crack.

**Figure 6:**
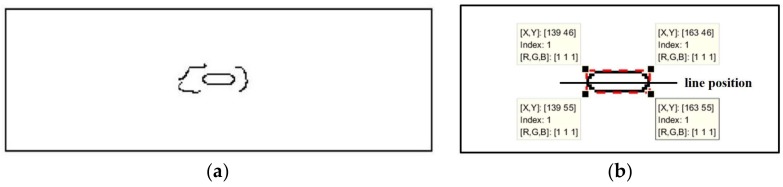
Edge detection results: (**a**) crack profile and (**b**) location of cracks and straight lines.

The edge detection result obtained by extracting the temperature data in Figure 5b is shown in Figure 6a. The shape of the crack is a rectangular approximately. As shown in Figure 6b, the circumscribed shape of the crack is a rectangle marked with red dotted line. The position of the crack in the two-dimensional array can be obtained by the circumscribed rectangle. The length of the crack is between the pixels 139 and 163, and the width is between 46 and 55, which is only a rough value.

To accurately explain the temperature variation, it was necessary to study the specific temperature distribution around the crack. A straight line parallel to the length of the crack is selected as the trajectory to better understand the temperature variation. In order to prevent errors caused by inaccurate crack boundaries, the straight line is preferably selected in the middle of the crack. Therefore, the line is placed at the 50rd pixel value, representing the crack width. The length of the line is 20 mm; its position is shown in Figure 6b.

**Figure 7:**
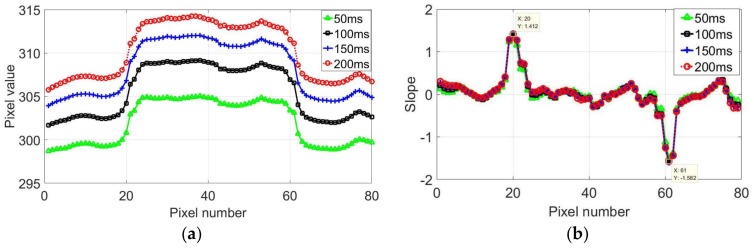
Data analysis of image: (**a**) temperature distribution of line at different times and (**b**) slope of first derivation at different times.

Figure 7a shows the temperature variation along the line for different times. The temperature of the crack area significantly changed during the heating process. A sudden change of the temperature at the crack boundaries was observed, and the entire crack corresponded to a high-temperature area. The temperature variations at different times were similar; however, with increasing heating time, the temperature difference became clearer, and the crack identification was easier. Figure 7b shows the slope curve of the temperature distribution along the line at different times. The slope curves at different times are exactly same, indicating that the slope curve is not affected by the heating time under a certain condition. The slope changes significantly when the temperature has a sudden change. The length of the crack can be determined by the distance between the maximum and minimum values on the slope curve, providing a theoretical basis for the quantitative evaluation of the cracks.

## 4. Experimental Verification and Evaluation

Based on the simulation results and theoretical analysis, it was shown that the temperature around a crack clearly increases during the heating process. For the experimental studies, an experimental ECPT system was setup, and the temperature at the crack boundaries was studied. The parameters and materials used for the experiments were selected based on the simulation analysis. Simultaneously, the quantitative evaluation method of the crack size was investigated.

### 4.1. Experimental System

**Figure 8:**
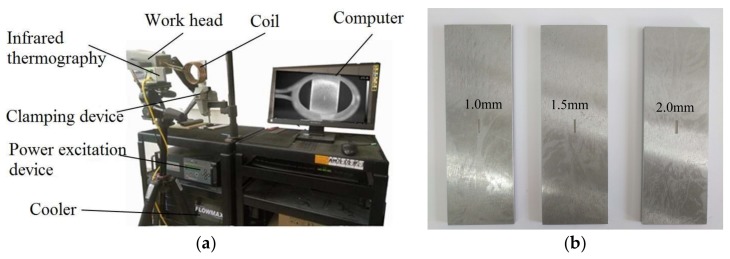
System design: (**a**) experimental ECPT system and (**b**) samples with different widths.

As shown in Figure 8a, the ECPT experimental platform included an infrared thermography system, a computer, an inductive heating device, coils, sample clamps, and other devices. An EASYHEAT 1.2–2.4-kW induction heating system AMBRELL (USA) was used for coil excitation, which had an excitation frequency range of 150–400 kHz, maximum excitation power of 2.4 kW, and maximum current of 400 A. A spiral coil is constructed to apply excitation. This coil is made of high conductivity hollow copper tube with the diameter of 8 mm, and water is pumped through the coil during operation to aid in cooling. In addition, the infrared thermography is FLIR T10xx with a 1024 × 768 image resolution. This camera has a sensitivity of <20 mK and a maximum full frame rate of 480 Hz.

In the experiment, 45# steel samples with different sized cracks on the surface were used as the detection objects. As shown in Figure 8b, consistent with the simulation model, the sample dimensions were 150 mm × 50 mm × 5 mm. The crack produced by artificial processing was located in the center of the sample to ensure that the collected temperature data were not missed. In this study, cracks with three different widths (1.0, 1.5, and 2.0 mm) were used with a constant length and depth of 10 and 0.6 mm, respectively.

### 4.2. Analysis of Experimental Results

**Figure 9:**
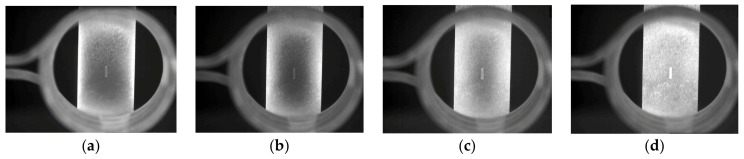
Thermal images after (**a**) 0.5 s, (**b**) 1 s, (**c**) 1.5 s, and (**d**) 2 s.

The sample with 2-mm width was selected as the experimental object. Based on the actual conditions and simulation results, the vertical distance from the infrared thermography system to the sample was set to 20 mm, the coil current was set to 380 A, and the excitation frequency was set to approximately 180 kHz, which was matched by the induction heating system automatically. During the experiments, thermal images were taken using the infrared thermography system. The results after heating for 2 s were selected for analysis and discussion. Images of the samples after 0.5, 1, 1.5, and 2 s are presented in Figure 9. The position of the coil and the sample can be observed clearly in the thermal image. With increasing heating, the surface of the sample gradually became bright, with the area around the crack being the most prominent. Bright areas in thermal images always indicate high-temperature regions, and the bright area around the crack was most obvious at 2 s. This result indicates that the eddy current accumulated in the cracks, resulting in gradual temperature increases around the cracks. Therefore, the cracks became the areas with the highest temperature in the sample, which corresponds well with the simulation results.

### 4.3. Quantitative Analysis of Cracks

**Figure 10:**
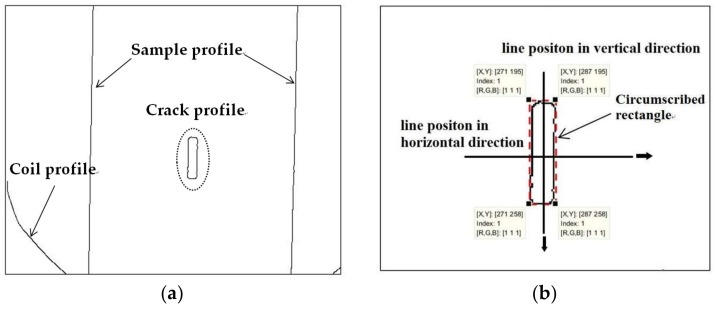
Edge detection results: (**a**) crack profile and (**b**) location of cracks and two straight lines.

The cracks and their locations were clearly observed in the thermal images; however, it was not possible to obtain accurate quantitative information about the crack geometry, i.e., its width and length, which are important parameters for accurate description of a crack. The thermal imaging image was processed by the edge detection algorithm, and the contour of the crack and sample were realized. As shown in the dashed box of Figure 10a, the profile of the crack is approximately rectangular. The rectangle shown in Figure 10b indicated by the dashed line is the circumscribed shape of the crack profile after the edge detection. Circumscribed rectangles can be used to represent cracks approximately. The length of the circumscribed rectangle is between pixels 195 and 258, and the width is between pixels 271 and 287. Since the shape of the crack is a rectangle, the trajectory selected for analyzing the temperature is two straight lines, which are along the horizontal direction and the vertical direction, respectively. Therefore, a line along the horizontal direction is selected at the 225rd pixel value representing the crack length, and another line along the vertical direction is selected at the 280rd pixel value representing the crack width. The distance between the pixels is unknown, so the two lines both pass through 200 pixels.

**Figure 11:**
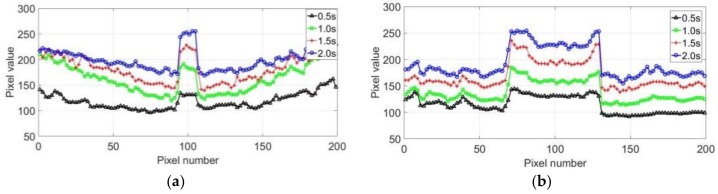
Temperature distribution of the line at different times: (**a**) horizontal and (**b**) vertical direction.

The pixel values obtained from the thermal images were used to represent the temperature distribution. Figure 11 shows the temperature distributions of two straight lines in the horizontal and vertical directions, which are indicated in Figure 10b. Besides, the 200 pixels in both directions are selected in the direction of the arrow in Figure 10b. The experimental results were mostly consistent with the simulation curves presented in Figure 7a. A sudden change in the temperature was detected in the crack boundaries, and the temperature variations became more apparent with increasing heating time.

**Figure 12:**
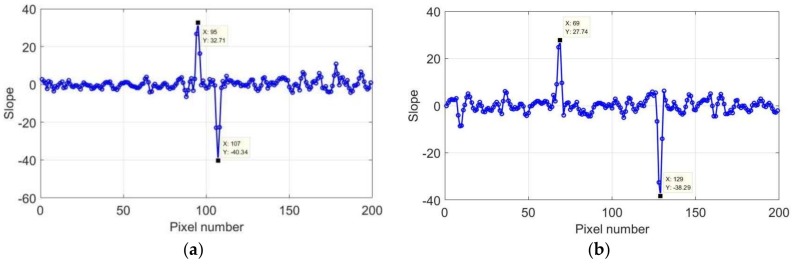
Slope of first derivation of 2 mm at 2 s: (**a**) horizontal and (**b**) vertical direction.

The length of the crack boundaries could be represented by the length of the high-temperature area. Therefore, the size of the rectangular crack could be determined by the number of pixels in the high-temperature region, and the areas where the sudden change was generated could be considered as the crack boundaries. In the horizontal direction, a significant temperature difference was observed between pixels 93–95 and 105–108. In the vertical direction, a sudden change was observed between pixels 68–71 and 129–132. It was difficult to accurately determine the locations of the boundaries, because the location of the sudden change spanned a few pixels. To reduce the data error, the first derivation of the data after 2 s was calculated, and the slope curve is presented in Figure 12.

Figure 12 shows that there was a large slope change of the crack boundaries in both directions, whereas the slope of the other part approached 0. The maximum and minimum of the slope could be used to locate the crack boundary. Figure 12a shows that the crack was present between pixels 95–107, and that pixels 95 and 107 represent the locations of the crack boundaries in the horizontal direction. Similarly, Figure 12b shows that the crack boundaries in the vertical direction can be represented by pixels 69 and 129. Therefore, the crack length corresponds to 60 pixels, with a width of 12 pixels.

**Figure 13:**
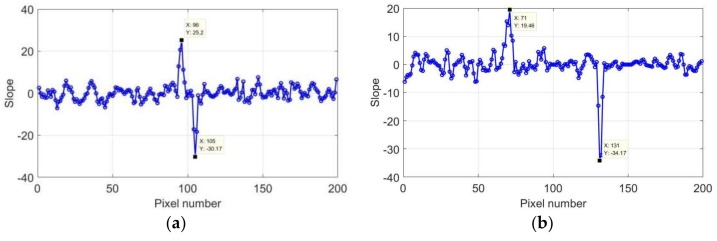
Slope of first derivative of 1.5 mm: (**a**) horizontal and (**b**) vertical direction.

**Figure 14:**
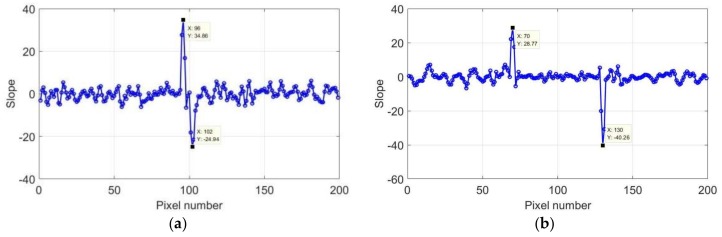
Slope of first derivation of 1.5 mm: (**a**) horizontal and (**b**) vertical direction.

Different cracks were employed to verify the reliability of the crack detection method, and the slope curves are presented in Figure 13 and Figure 14. Two lines crossing the crack along the horizontal and vertical directions were selected.

The results with widths of 1.5 mm and 1.0 mm after a heating duration of 2 s were selected for the analysis and discussion. When the crack width was 1.5 mm, the crack parts were located at pixels 96–105 in the horizontal direction and pixels 71–131 in the vertical direction. The crack length corresponded to 60 pixels, and the crack width was 9 pixels. When the crack width was 1.0 mm, the crack parts were located at pixels 96–102 in the horizontal direction and at pixels 70–130 in the vertical direction. The crack length corresponded to 60 pixels, and the crack width was 6 pixels.

**Figure 15:**
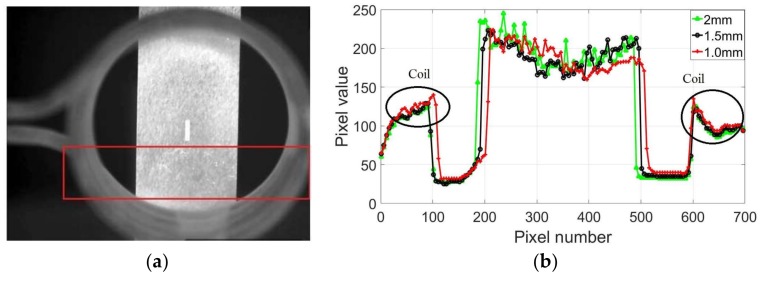
Data analysis of image: (**a**) non-crack area and (**b**) temperature distribution of line of three samples with different widths.

In the slope curve, the number of pixels that respectively represent the length and width of the cracks can be determined, and the distance between two pixels needs to be determined to calculate the specific size of the crack. Since the width of the sample in the experiment is a known value, the distance between two pixels can be determined by determining the number of pixels representing the width of the sample. In Figure 10, the boundaries of the sample and crack can be observed. The pixel value of the crack area is determined, resulting in the pixel value of the non-crack area also being determined. As shown in Figure 15a, a straight line in the horizontal direction is selected in the non-crack area of the red rectangle in the thermal image based on the pixel value of the non-crack area, and the temperature along the straight line is analyzed. The positions of the straight lines in the horizontal direction were selected from the three different samples, and the temperature distribution was analyzed. The results are shown in Figure 15b.

**Figure 16:**
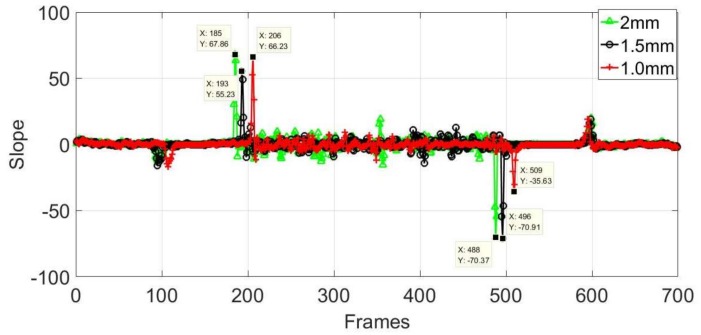
Slope of first derivation of three different samples.

As shown in Figure 15b, the temperature distributions of the non-crack area for the three different samples are basically similar. The higher temperature part on both sides is the coil temperature, and the highest temperature area in the middle indicates the temperature distribution of the sample surface, which is similar to the temperature distribution of the crack area. The temperature has a sudden change at the boundaries of the sample. It is difficult to ensure that the samples are placed in the same position in each experiment, so the three temperature distribution curves indicate the coil, and the sample cannot be completely overlapped. The first derivation of the temperature was calculated for more accurate results, and the slope curves are presented in Figure 16.

In Figure 16, the pixel points corresponding to the maximum and minimum slope indicate the boundaries of the samples, and the number of pixels indicating the width of the samples in the three curves is 303. The width (50 mm) of the sample corresponded to 303 pixels, so the distance between each two pixels can be calculated. The number of pixels representing widths and lengths of different cracks has been determined so that the actual size of the crack can be determined. The results for the three groups are compared with the actual sizes in Table 2.

As shown in Table 2, the experimental errors of the three groups were close to 1%. Because the same crack length was used, the calculated values obtained in the three groups were same, and all the length errors were 0.99%. The differences in the width errors were small. It can be concluded from the experimental results that the crack sizes of the samples were precisely determined. Therefore, quantitative crack detection can be performed using the proposed analysis method.

## 5. Conclusions

In this paper, the use of ECPT for crack detection based on temperature analysis was investigated using simulation and experimental studies. A quantitative crack detection method was proposed based on derivative analysis, and cracks of different sizes were quantitatively analyzed. The main contributions of this work include the following conclusions:

(i) For ECPT testing, reducing the distance between the coil and sample surface and increasing the coil current and excitation frequency can improve the heating efficiency; this information can be combined with actual experimental conditions for selection of the appropriate lift to ensure a better heating effect.
(ii) Cracks are located in the regions where the temperature and eddy current density are the highest. Thus, the crack profile in the thermal image can be extracted by the Canny algorithm to determine the shape and position of the crack. The crack geometry size is determined by the temperature distribution of crack trajectory.
(iii) Crack boundaries can be represented by the pixels corresponding to abrupt changes of the surface temperature, and the crack size trajectory can be determined based on the number of pixels between the two boundaries. In the experiments, the crack size was determined by analyzing the slope curve, and the length and width errors of the cracks were no higher than 1%, verifying the reliability of this detection method. These findings are significant for quantitative crack detection based on ECPT.

## Figures and Tables

**Table 1:** Properties of steel.

	Density	Thermal Conductivity	Heat Capacity	Electrical Conductivity	Relative Permeability
**steel**	7850 [kg/m^3^]	44.5 [W/(m × K)]	475 [J/(kg × K)]	1.032 × 10^6^ [S/m]	1

**Table 2:** Experimental results of three groups.

Actual Size (mm)	Calculated Size (mm)		Length Error	Width Error
	Length	Width		
10 × 2.0	9.901	1.9802	0.99%	0.990%
10 × 1.5	9.901	1.4851	0.99%	0.993%
10 × 1.0	9.901	0.9900	0.99%	1%

## References

[1] Takahashi S., Kobayashi S., Tomáš I. (2017). Comparison of magnetic nondestructive methods applied for inspection of steel degradation. NDT E Int..

[2] Siakavellas N.J. (2016). The Influence of the heating rate and thermal energy on crack detection by eddy current thermography. J. Nondestr. Eval..

[3] Hellier C. (2001). Handbook of Nondestructive Evaluation.

[4] Li J.W., Chen J.H. (2002). Handbook of Nondestructive Testing.

[5] Xu K.B., Zhou J.H. (2004). Eddy Current Testing.

[6] Ren J.L., Lin J.M. (2008). Electromagnetic Nondestructive Testing.

[7] Pan M.C., He Y.Z. (2013). Eddy Current Thermography Non-Destructive Testing.

[8] Fu J., Lei Y. (2016). Study on sinusoidal eddy current test method for ferromagnetic plate parameters. Chin. J. Sci. Instrum..

[9] Garnier C., Pastor M.L., Eyma F. (2011). The detection of aeronautical defects in situ on composite structures using non-destructive testing. Compos. Struct..

[10] Chen X., Lei Y. (2014). Excitation current waveform for eddy current testing on the thickness of ferromagnetic plates. NDT E Int..

[11] Tian G.Y., Gao Y., Li K. (2016). Eddy current pulsed thermography with different excitation configurations for metallic material and defect characterization. Sensors.

[12] Wilson J., Tian G.Y., Mukriz I. (2011). PEC thermography for imaging multiple cracks from rolling contact fatigue. NDT E Int..

[13] Zhou X., Zhou J., Tian G.Y. (2015). Research on defects inspection of solder balls based on eddy current pulsed thermography. Sensors.

[14] Baek S., Xue W., Feng M.Q. (2012). Nondestructive corrosion detection in RC through integrated heat induction and IR thermography. J. Nondestr. Eval..

[15] Chen H.Y., Tao H.L. (2009). The Characteristics and application selection of common nondestructive testing methods. Nondestr. Test..

[16] Oswald-Tranta B. (2004). Thermoinductive investigations of magnetic materials for surface cracks. Quant. Infrared Thermogr. J..

[17] Weekes B., Almond D.P., Cawley P. (2012). Eddy current induced thermography probability of detection study of small fatigue cracks in steel, titanium and nickel-based superalloy. NDT E Int..

[18] Noethen M., Yi J., Meyendorf M. (2012). Simulation of the surface crack detection using inductive heated thermography. Nondestr. Test. Eval..

[19] Cheng L., Tian G.Y. (2012). Comparison of nondestructive testing methods on detection of delaminations in composites. J. Sens..

[20] Yang S., Tian G.Y., Abidin I.Z. (2011). Simulation of edge cracks using pulsed eddy current stimulated thermography. J. Dyn. Syst. Meas. Control.

[21] Abidin I.Z., Tian G.Y., Wilson J. (2010). Quantitative evaluation of angular defects by pulsed eddy current thermography. NDT E Int..

[22] He Y.Z., Tian G.Y., Pan M. (2013). Eddy current pulsed phase thermography and feature extraction. Appl. Phys. Lett..

[23] Li T.Y., Tian Y.P., Sun R., Wang P. Simulation of cracks detection using pulsed eddy current thermography. Proceedings of the 2012 Far East Forum on Nondestructive Evaluation/Testing: New Technology, & Application.

[24] Zhu P., Yin C., Cheng Y. (2017). An improved feature extraction algorithm for automatic defect identification based on eddy current pulsed thermography. Mech. Syst. Signal Process..

[25] Bai L.B. (2013). Research on Nondestructive Testing Technology of Dddy Current Pulsed Thermography.

[26] Xu H.K., Qin Y., Chen H.R. (2014). An improved algorithm for edge detection based on Canny. Infrared Technol..

[27] Li K., Tian G., Cheng L., Yin A., Cao W., Crichton S. (2013). State detection of bond wires in IGBT modules using eddy current pulsed thermography. IEEE Trans. Power Electron..

